# Sleep Posture and Hydration: Simple Lifestyle Interventions With Therapeutic Cardiovascular Health Potential?

**DOI:** 10.1016/j.jacadv.2024.101539

**Published:** 2025-01-08

**Authors:** Mohammed Y. Khanji, C. Anwar A. Chahal, Shahid Karim

**Affiliations:** a William Harvey Research Institute, NIHR Barts Biomedical Centre, Queen Mary University of London, London, UK; b Newham University Hospital, Barts Health NHS Trust, London, UK; c Department of Cardiology, Barts Heart Centre, St. Bartholomew’s Hospital, Barts Health NHS Trust, London, UK; d Department of Cardiology, Mayo Clinic, Rochester, MN, USA; e Center for Inherited Cardiovascular Disease, WellSpan Cardiology, New York, PA, USA

**Keywords:** cardiac physiology, cardiovascular health, hydration, lifestyle interventions, sleep posture

**Figure:**
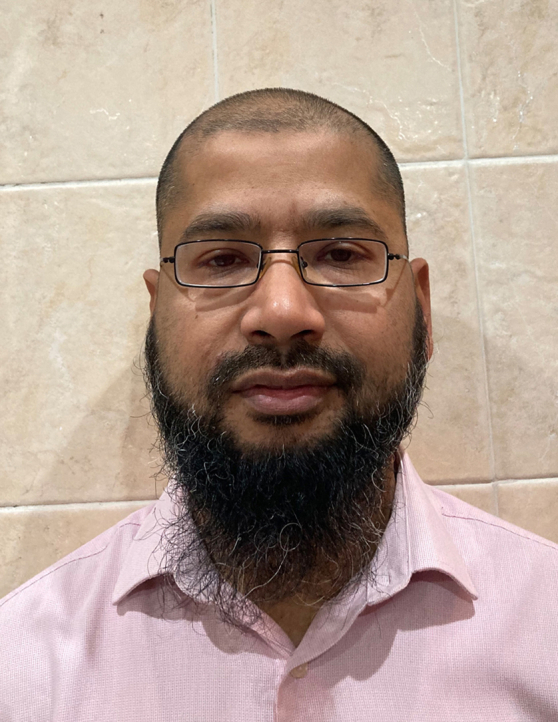

The cardiovascular implications of fluid regulation have been extensively debated. In the late 19th century, the neurologist Sir William Gowers systematically described orthostatic hypotension as a clinical phenomenon involving a significant drop in blood pressure upon standing.^1^ In 1940, Maclean and Allen published the concept of positional therapy, including head-up-tilt, as a treatment for orthostatic intolerance.^2^ Prior studies have primarily centered on hydration in acute or exercise contexts, leaving a gap in the understanding of medium- to long-term impacts of systematic fluid intake increases. Moreover, head-up sleep (HUS)—a modification of nocturnal posture—has traditionally been explored in patients with orthostatic intolerance but not in the general population.

Guo et al's informative study on the “Medium-Term Effects of Increased Water Intake and Head-Up Sleep on Cardiovascular Health” provides a novel perspective on elementary lifestyle interventions targeting cardiovascular health, addressing some of these gaps.^3^ Thus, aligning with a growing interest in the role of everyday lifestyle adaptations in enhancing cardiovascular resilience. The study evaluates and compares the effects of solitary increased fluid intake (IFI), against its combination with HUS (IFI + HUS) on cardiac structure, function, and aerobic capacity in healthy adults. Importantly, the study contributes to the broader understanding of nonpharmacological strategies aimed at optimizing cardiac function, with implications for both clinical practice and future research directions.

Key findings from the study reveal that while neither IFI nor IFI + HUS altered intravascular volumes nor aerobic capacities, they induced measurable improvements in cardiac performance at rest. Both interventions increased left ventricular end-diastolic volume and stroke volume, reduced arterial elastance, and improved diastolic relaxation (notably more pronounced with IFI + HUS). These structural and functional adaptations, occurring without changes in peak exercise capacity, suggest mechanisms independent of blood volume expansion.^4^ The absence of significant changes in aerobic capacity contrasts with previous studies linking fluid and blood volume modulation to enhanced oxygen delivery and consumption. This divergence underscores the tight regulatory balance of the cardiovascular system, particularly in healthy populations with intact fluid and electrolyte homeostasis.

These findings hold translational potential, especially for populations at risk of cardiac deconditioning or suboptimal hemodynamics. The reported improvements in resting cardiac function, from this relatively small sample, may inform noninvasive, lifestyle-based interventions for conditions such as mild heart failure or orthostatic intolerance. Notably, HUS, a simple intervention, may be particularly beneficial in populations where pharmacological strategies are either contraindicated or poorly tolerated. However, caution is warranted before extrapolating these results to clinical cohorts, particularly where fluid management is critical, such as in patients with decompensated heart failure.

Guo et al's study highlights several avenues for further inquiry. First, exploring these interventions in patient populations with altered hemodynamic regulation, such as heart failure with preserved ejection fraction or other causes of dysautonomia, may provide insights into their therapeutic potential. Second, mechanistic studies investigating neurohormonal pathways, including the renin-angiotensin-aldosterone system and natriuretic peptide dynamics, might elucidate the underlying processes driving the adaptations reported.

Third, integrating wearable technology to monitor adherence and physiological responses in real-time could be explored to assess reliability and scalability of these interventions. Finally, longitudinal studies extending beyond 3 months, beyond healthy controls and with larger sample size would help determine whether these cardiovascular phenotype and functional changes translate into improved quality of life or clinical outcomes, and which patients groups may gain most benefit.

In summary, this work by Guo et al underscores the nuanced interplay between hydration, posture, and cardiovascular health. By elucidating how simple, cost-effective interventions can optimize cardiac function in healthy individuals, this study lays a foundation for innovation in preventive and therapeutic cardiology, challenging researchers and clinicians to think creatively about harnessing physiological adaptations through lifestyle interventions to improving cardiovascular resilience across diverse populations.

## Funding support and author disclosures

The authors have reported that they have no relationships relevant to the contents of this paper to disclose.
